# Decisions about risk taking: Elaborate dynamics between guests and hosts of peer-to-peer accommodation during COVID-19

**DOI:** 10.1371/journal.pone.0341733

**Published:** 2026-03-16

**Authors:** Anan Hu, Jinyuan Pang, Xuena Gan

**Affiliations:** 1 Department of Tourism, Fudan University, Shanghai, China; 2 School of Tourism, Anshun University, Anshun, Guizhou, China; Universiti Malaya, MALAYSIA

## Abstract

This study explores the influence of COVID-19 on peer-to-peer accommodation, from perspectives of both market performance and guests’ mindset. Combination of qualitative and quantitative data, as well as analysis by smaller time unit, enables more subtle insights into participants’ behavior. Based on Event System Theory, authentic market data is examined to demonstrate the trend of demand and supply of Airbnb housing in New York City. Online reviews are investigated, using Structural Topic Model, to reveal how guests’ focuses transformed. Results show that peer-to-peer accommodation was stricken heavily by COVID-19 and subsequent events, where booking volume shrank more severely, and reacted faster to exogenous environment, compared to housing supply. Guests shifted their attention after COVID-19 happened, caring more about hygiene and social distance. The synchronization of fluctuation among multiple variables validates the assumption that exogenous events successively influenced guests’ attitude and market performance. Apropos of events, stronger incidents exhibited larger influence on market performance, and event timing affected exogenous events’ influence upon the industry. This research enriches Event System Theory and adds to the insights into elaborate dynamics among participants of peer-to-peer accommodation industry during COVID-19. The findings provide hospitality practitioners and governments with reference for future risk management.

## Introduction

The COVID-19 pandemic exerted a huge and profound impact worldwide, disrupting nearly every aspect of society and triggering the largest global economic crisis in decades [1,2]. The hospitality industry, particularly its peer-to-peer (P2P) accommodation segment, was exceptionally vulnerable. As a burgeoning sector poised to disrupt the traditional lodging industry, where platforms like Airbnb boasted more listings than the top five hotel chains combined, the pandemic introduced an unprecedented shock that caused bookings and revenues to plummet [3]. According to the 2020 annual report of Airbnb, Inc., number of nights and experiences booked on Airbnb fell from 327 million in 2019–193 million in 2020, and the gross booking value suffered a 37% YOY drop [4].

Studies have reached into several aspects of how the pandemic has influenced peer-to-peer accommodation. Compared to shared rooms, entire apartments became more favored owning to their advantage in social distancing [5]. An investigation into COVID-19 Stringency Index and Airbnb data has shown that strong response policies about the pandemic causes decline in Airbnb housing demand [6]. When it comes to guests’ mindset, guests’ reviews on Airbnb have revealed that tourists’ feelings and thoughts also went through disturbance [7]. Despite these advances, critical gaps remain. Most existing literature examines objective market data and subjective guest perceptions in isolation, failing to trace how external events shape both behaviors and mindsets simultaneously [8,7]. Furthermore, insufficient attention has been paid to the dynamic, time-sensitive nature of risk perception, with most studies adopting simple “before-and-after” comparisons that overlook crucial fine-grained fluctuations. While Event System Theory (EST) is relevant, there is limited understanding of how specific COVID-19–related events—defined by their strength and timing—systematically translate into risk perceptions and behavioral adjustments for different platform participants. Consequently, while the vulnerability of P2P market is displayed, we still lack a systematic, event-based explanation connecting the evolving health threat to the elaborate dynamics of the sharing economy.

To address these issues, this study positions Event System Theory as its core lens to investigate the impact of COVID-19 on peer-to-peer accommodation in New York City during 2020. In this study, market performance is specifically operationalized through two key quantitative indicators: booking volume (representing market demand) and the volume of active listings (representing market supply). Unlike previous research, this paper synthesizes this quantitative data with qualitative data (guests’ online reviews analyzed via Structural Topic Model) using a fine-grained time unit. By doing so, this research aims to construct a solid logic chain connecting outside events to consumers’ mindsets, and ultimately to market behavior, providing a more inclusive insight into how a major health threat influences the multiple parties of this industry.

Specifically, two questions are to be answered:

1. How was the market performance affected by COVID-19-related events?
2. Did guests’ foci change after COVID-19 took place?

By answering these questions, this article enriches knowledge about the P2P accommodation industry during the COVID-19 pandemic in four folds:

1. Quantitative data (market demand and supply volume) and qualitative data (guests’ online reviews) are synthesized to investigate the comprehensive industry, while most prior literature employ only one of these aspects. Structural Topic Model is used to analyze online reviews.
2. Behaviors of both guests and hosts in peer-to-peer accommodation market are studied, which leads to more inclusive insight of how a health threat influences multiple parties of an industry.
3. Factors are analyzed in smaller time unit, revealing more elaborate dynamics during different stages. Former researchers conducted before-and-after or monthly comparison, while this paper includes daily reviews data with more details.
4. Based on Event System Theory, this paper categorizes the incidents relevant to the pandemic and rates their strength, therefore able to investigate their influence in a more systematic approach and provide practical implications for risk management in the future.

This paper is structured as follows: The Literature Review section introduces the theoretical background and previous researches related to this topic. The Proposition Development section presents the design and hypotheses of the study. The Materials and Methods section explains the methods and data to be used, and the Results section reports the results of the analysis. The Discussion section addresses the discussion and implications, and the Conclusions section presents the final conclusions of the study.

## Literature review

### Tourism, accommodation, and consumer behavior during COVID-19

All sections that involve travels suffered severe damage from COVID-19, since movement and gathering became risky and troublesome. Tourism, accommodation, restaurant are typical cases. A study employing Google-released movement trend data shows that people’s out-of-home social interaction was reduced by COVID-19-related policies, and the extent of impact varies according to policy types [9]. In hotel industry, guests’ satisfaction level around the world notably dropped after the pandemic happened [10]. Perceived hygiene attributes have been proven to have significant influence on hotel image and word of mouth during the pandemic [11]. Reassuring crisis communication and lower level of interaction can raise customers’ booking intention, where rational appeal type of marketing communication messages generates larger positive influence than emotional appeal type [12,13].

In peer-to-peer accommodation industry, COVID-19’s influence has been investigated. Market-wise, decrease in demand, supply, and price has been widely confirmed, especially in famous tourist attraction cities [14,5,15,16]. Meanwhile, some cities actually saw a raise in rental volume, which might result from increased demand for domestic travel and encouraging travel policies [5]. Overall, the industry suffered huge damage. After a set of calculation based on booking volume, length of stays and housing price, it’s concluded that Airbnb hosts in Greater Sydney suffered a nearly 90% income loss due to the pandemic [8]. Within the demand end of the market, travelers’ thoughts have been revealed by their preference. When tourists expect higher threat of COVID-19, they become less willing to use Airbnb accommodation [17]. Several research have discovered tourists’ preference towards full flats over shared flats or over hotels during the pandemic [5,18]. People’s foci and preferences have shifted, paying more attention to sanitary, favoring dwellings with price premium, and reducing host-guest interactions [19–22]. These demonstrate customers’ concerns about physical distance and hygiene during the pandemic.

Among accommodation hosts arises heterogeneity. Price adjustments and flexible cancellation policies have been employed to appease Airbnb guests [23]. Compared to non-professional hosts, professional hosts in Barcelona lower their prices to a greater extend due to COVID-19, which suggests their proficiency at practicing price discrimination [24]. Interviews unveil the attitude of hosts around the world during the pandemic, optimistic ones updated strategies to continue their business while pessimistic ones felt disappointed and decided to quit the market [25].

### Event system theory

Event system theory (EST) stands out as a highly applicable framework for analyzing the tourism and accommodation industries, which are known for their vulnerability to exogenous incidents. EST bridges two major types of organizational science theories—variance theories, focusing on stable features, and process theories, focusing on dynamic influences [26]. This creates an integrative framework to investigate not only the stable characteristics of entities but also the dynamic impact of events [27].

**Fig 1:**
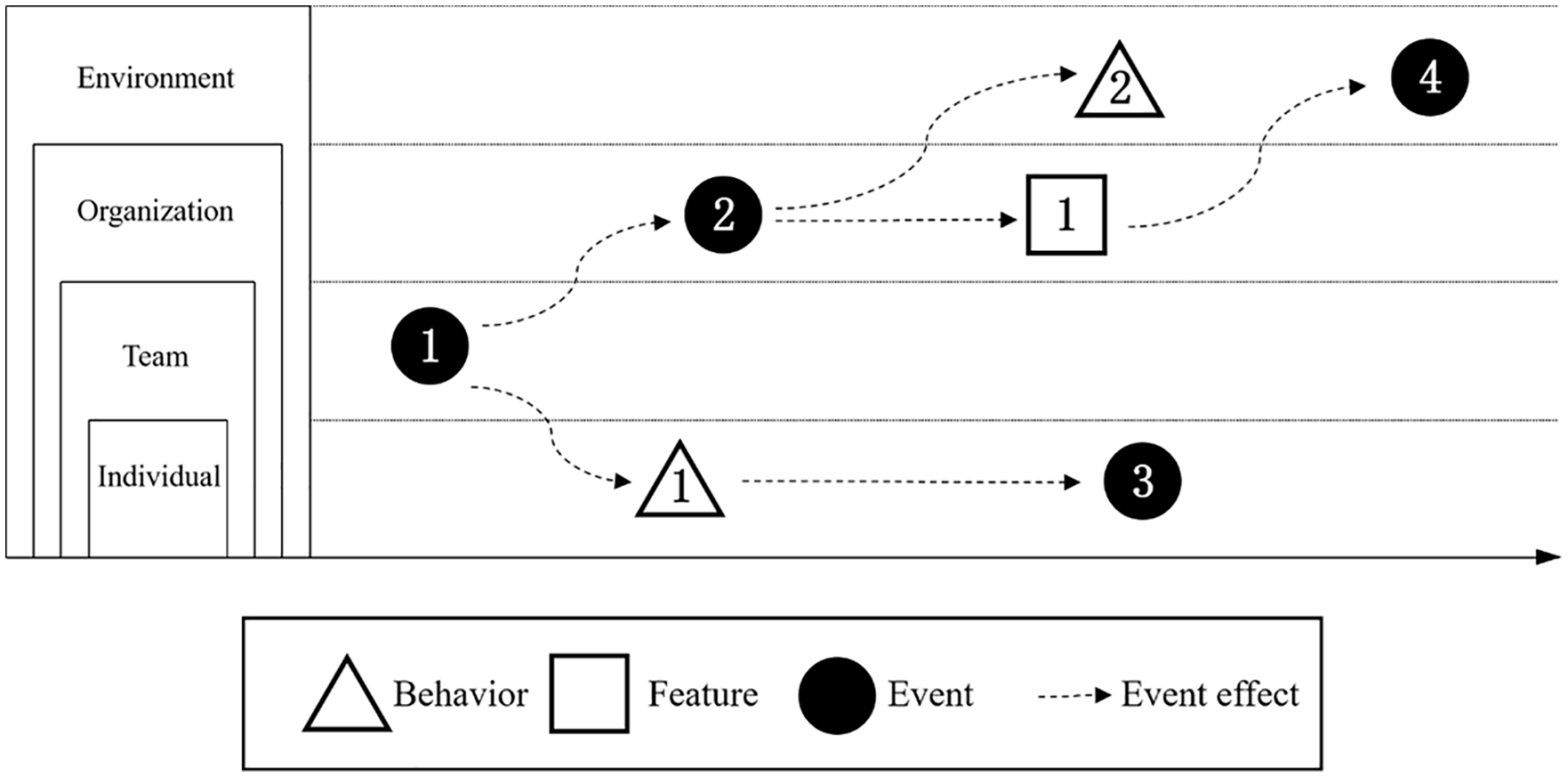
Effects of Events on Entities.

The core of EST is its analysis of how events influence entities (e.g., individuals, teams, organizations) over time, based on three primary characteristics [28]. The first is event strength, which is determined by an event’s novelty, disruption, and criticality; a stronger event is more likely to cause significant change. The second characteristic, event space, refers to an event’s origin and the pathways of its influence, including factors like spatial direction, dispersion, and proximity, which moderate the event’s ultimate impact. Finally, event time considers an event’s duration, its specific timing, and any changes in its strength over its course [29]. By analyzing these characteristics, EST directly addresses the core questions in major event research: “how impacts occur, how they diffuse, and how they evolve,” providing a basis to identify impact pathways and predict long-term consequences [27]. Fig 1 illustrates some general forms of events’ effects on organizations [27].

The applicability of EST is particularly potent for analyzing systemic shocks like the COVID-19 pandemic. Such occurrences are not monolithic but are a cascade of discrete events whose specific characteristics dynamically shape outcomes, moving analysis beyond simple “before-and-after” comparisons.

Since the pandemic’s onset, EST has been effectively employed in related studies. For instance, research has shown that the pandemic’s strength significantly influenced employee job burnout, work connectivity, and emotional exhaustion in China [30–33]. It has also been used to analyze shifts in job-searching behavior and student anxiety [34,35]. These precedents, combined with EST’ s ability to model complex interactions in the tourism industry, confirm its suitability for this study.

Therefore, this paper adopts the EST framework to investigate the peer-to-peer accommodation industry during COVID-19. Within the framework of this study, these entities are specifically defined as follows: the ‘individual’ entity is represented by Airbnb supply; the ‘team’ entity corresponds to Airbnb booking; the ‘organization’ is conceptualized as the series of key events related to COVID-19; and the ‘environment’ refers to the overall state of the pandemic.

### Structural topic model for Text analysis

With massive quantity of customers’ reviews in tourism industries available, text analysis has become widely adopted for delving into travelers’ thoughts and feelings. Topic modeling, based on word counts of the corpus, is a prevalent approach. A corpus may contain multiple documents, and each document may contain numerous words. Bayesian techniques are employed in topic models to determine the topics within the corpus and words closely associated with the topics. By analyzing groups of words together instead of each word individually, topic modeling can reach a result that’s closer to the meaning of words in the context of natural language [36–38].

Structural topic model (STM) is one of the topic models. Proposed in 2013, it has integrated metadata about documents into the model, thus able to examine relationships between topics and covariates. This achievement fills the gap left by another popular topic model of Latent Dirichlet allocation (LDA) [36].

Owning to its ability to analyze the changes of topics over time and various other advantages, STM is employed in this research to analyze online reviews on home-sharing platform and probe into travelers’ thoughts and foci.

**Fig 2:**
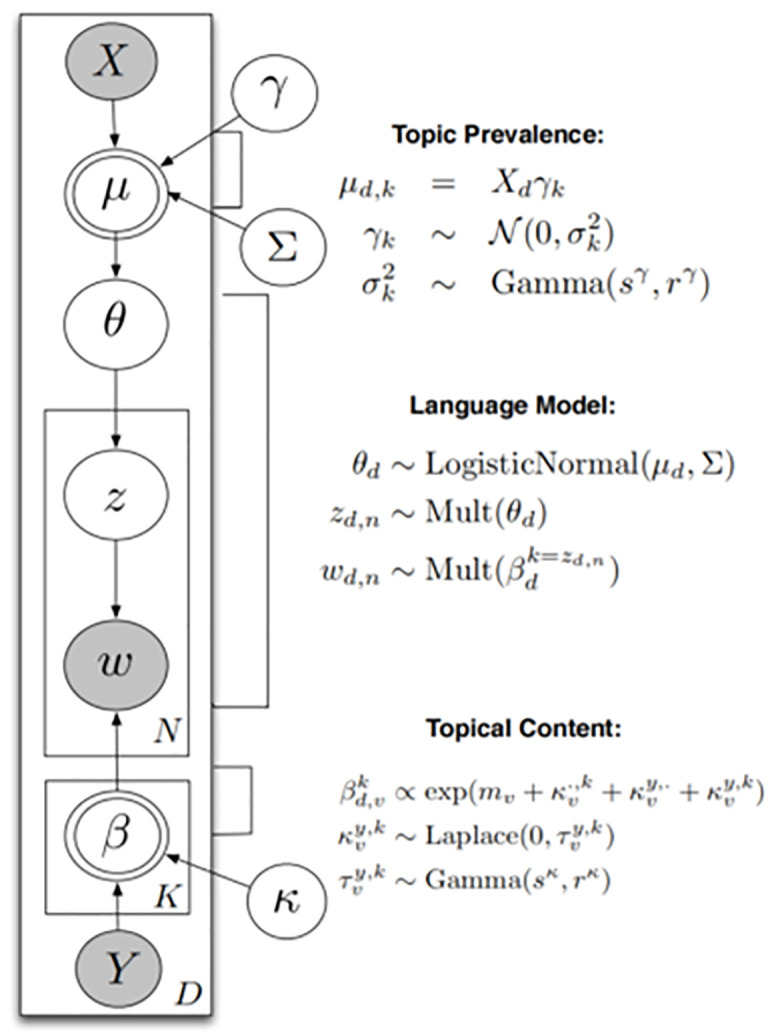
Plate diagram for the structural topic.

When constructing an STM model with *k* topics, each document in the corpus (indexed by *d*) with vocabulary of size *V* is processed in the following steps, as illustrated in Fig 2 [37,38]:

From a logistic-normal generalized linear model based on a vector of document covariates *X*_*d*_, document-level attention to each topic is allocated.


$$\theta \sim_{d}|X_{d\gamma },\;\sum \sim \text{LogisticNormal}(\mu \;=\;X_{d\gamma },\Sigma )$$ (1)

As the document-level content covariate *y*_*d*_ is set, a document-specific distribution over words representing each topic (*k*) is formed. The calculation involves baseline word distribution (*m*), the topic specific deviation *k*^*(t)*^_*k*_, the covariate group deviation *k*^*(c)*^_*yd*_, and the interaction between the two *k*^*(i)*^_*yd,k*_.


$$\beta_{d,k}\propto \text{exp}(m+k_{k}^{\left(t\right)}+k_{yd}^{\left(c\right)}+k_{yd,k}^{\left(i\right)})$$ (2)

For each word in the document, (*n*∈{1,...,*N*_*d*_})

a Each word’s topic assignment is drawn based on document-specific distribution over topics.

$$z_{d,n}|\theta \sim_{d}\sim Multinomial(\theta \sim_{d})$$ (3a)

b Observed words from specific topics are drawn.

$$w_{d,n}|z_{d,n},\beta_{d,k=zd,n}\sim Multinomial(\beta_{d,k=zd,n})$$ (3b)

Eventually, a model consisting of *k* topics is produced. Detailed data such as topical content, topical prevalence, and most representative documents are also generated. Topical content is defined by the words that are closely associated with a given topic. Four versions of word profiles can be produced using different methods, which are highest probability, FREX, lift, and score. Topical prevalence is the proportion of document in the corpus that is devoted to the topic. Larger the proportion, more prevalent the topic [37,38].

STM has been previously used in research related to tourism settings, such as comparing popular topics and sentiments between Airbnb and hotel guest reviews [39]. After COVID-19, scholars have applied it to inquire people’s thoughts along the development of the pandemic. Topics of German twitters during the pandemic exhibit dominance of problem-focused content over emotion-focused content [40]. Online posts related to COVID-19 have revealed increasing concerns about job issues over time as prolonged situation starting to cause job-related problems, as well as disparities across ethnic and racial communities [41,42]. The model is also employed to trace how topics of COVID-related news reports and press briefings have changed as the pandemic went by [43,44].

## Proposition development

While COVID-19’ s influence on peer-to-peer accommodation has been looked into, previous literature seldom combines market performance and travelers’ thoughts to explore the full picture of the industry. The usual adoption of before-and-after comparison and monthly data also leaves an opening for more nuanced approach enabled by thinner time interval. This research is designed to fill in these gaps.

The study consists of two parts, one about market and the other about mindset, and to each one several propositions are made.

The following propositions are derived from Event System Theory and prior literature. Although we term them propositions to emphasize their exploratory and theory-building role, they are empirically examined in this study using market data and textual analysis to make the underlying logic explicit.

***Part one***: ***The impact of the pandemic on the peer-to-peer accommodation market’s booking and supply.***

Volumes of booking and supply are the most crucial indicators of lodging business. Since the pandemic discourages travel, it is expected that booking volume will decline, subsequently causing supply volume to decline as well. According to EST, stronger key events exert a larger influence on entities. During the pandemic, the strength and density of events varied significantly. Drawing on these principles, the following propositions are made:

**Proposition 1 (P1).** *During the pandemic, when COVID-19-related key events are most numerous, most frequent, and strongest, peer-to-peer accommodation booking volume undergoes the highest level of decline.*

**Proposition 2 (P2).** *During the pandemic, when COVID-19-related key events are most numerous, most frequent, and strongest, peer-to-peer accommodation supply volume undergoes the highest level of decline.*

**Proposition 3 (P3).** *During the pandemic, strength of COVID-19-related key events has a negative influence on peer-to-peer accommodation bookings volume.*

Extra investigation is put into types of listings, where a listing is a unit of housing to be rented on peer-to-peer platforms. Common types of listings on the market include an entire house/apartment, a single private room, and a room shared with others. Naturally, this leads to different level of isolation. Under the influence of COVID-19, people tend to keep adequate social distance to lower the risk of infection. The following propositions are therefore raised:

**Proposition 4a (P4a).** *During the pandemic, less isolated listings, where social distant are hard to maintain, experience larger degree of changes in booking volume.*

**Proposition 4b (P4b).** *During the pandemic, less isolated listings, where social distant are hard to maintain, experience larger extent of changes in supply volume.*

P1 through P4 are to be tested based on development of the pandemic, related exogenous events, and booking and supply data of peer-to-peer accommodation market.

***Part two***: Analyzing shifts in guests’ focus during the pandemic using STM on online reviews.

After COVID-19 hit, guests likely pay more attention to sanitary condition and social distance. These thoughts, as well as other foci of the traveler, are often revealed in their online reviews. Using STM, this research analyzes the posts they wrote, identifies their foci on lodging experiences, and examines the synchronization between shift of guests’ foci and development of the pandemic. The propositions are as follows:

**Proposition 5 (P5).** *After COVID-19 hit, guests of peer-to-peer accommodation pay more attention to sanitary condition, social distance, and other COVID-19-related issues than pre-pandemic time. The rise of this attention synchronizes with development of the pandemic.*

**Proposition 6 (P6).** *During the pandemic, the extent of changes in guests’ attention to sanitary condition, social distance and other COVID-19-related issues vary among different types of listings. Larger extent of changes in attention is found among guests of less isolated listings where social distance is harder to maintain.*

Propositions 5 and 6 are to be tested based on development of the pandemic, related exogenous events, and online review texts on peer-to-peer accommodation platform.

**Fig 3:**
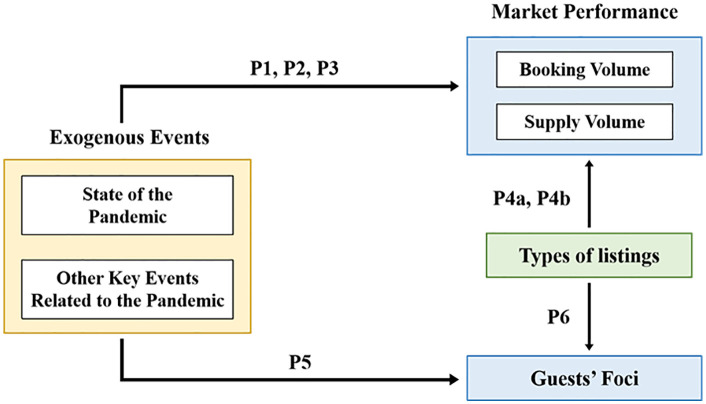
Framework of the Study.

With these two parts combined, the study shall develop an insight into the elaborate dynamics among various factors in the specific background. Fig 3 exhibits the framework of this study.

## Materials and methods

### Space and time range

This study investigates peer-to-peer accommodation during the pandemic. Space and time range of the object are limited for the research to be better focused.

*Space-wise*, peer-to-peer accommodation in New York City is chosen as the target of analysis for several reasons. First, NYC is one of the most influential tourism destinations, welcoming over 66 million global tourists in 2019 [45]. It was also among the cities in the U.S. that are stricken the hardest during 2020. Third, with administratively defined boundaries, situation of the pandemic and COVID-19-related policies are usually consistent within this region, which makes “city” an appropriate unit. Also, NYC offers abundant public, official, and relatively reliable data about the pandemic. These combined make NYC a suitable case for this study.

*Time-wise*, instead of covering the whole several years affected by COVID-19, this study investigates the data of the year 2020. The prime reason is that being the first year of the pandemic, 2020 is the most representative. In NYC, as well as many other regions, people experienced the first wave of COVID-19, temporary recovery, and a second wave, all in 2020. People’s common mindset went from ignorant, to nervousness, then to a mixture of denial, persistence, and tiresome. Later course of the pandemic was mostly similar to that of 2020. The second reason is that a duration of one year is more feasible for this study than a lengthier period. This paper goes through detailed data by thin time interval; therefore an overextended span might compromise the analysis of its subtlety and concentration.

### Data collection

Table 1 exhibits the data used for this study. The two main categories are exogenous events and peer-to-peer accommodation data. Exogenous events are further divided into two subcategories: the state of the pandemic itself, and other key events related to COVID-19. The peer-to-peer accommodation data consists of booking, supply, and online reviews by guests.

**Table 1:** Data for this Study.

Main category	Subcategory	Source of data	Definition/Unit	Time Period
Exogenous Events	State of the pandemic	NYC Department of Health and Mental Hygiene	Number of daily new COVID-19 positive cases in NYC. (Unit: cases per day)	2020/1/1 - 2020/12/31
	Key events related to COVID-19	Multiple sources	Discrete happenings (e.g., policy announcements) rated on a 3–9 strength scale. (Unit: event instance)	2020/1/1 - 2020/12/31
Peer-to-peer Accommodation Data	Market performance – booking	Inside Airbnb	Volume of online reviews used as a proxy for booking volume. (Unit: reviews per day)	2019/1/1 - 2020/12/31
	Market performance – supply	Inside Airbnb	Number of active listings available on the platform. (Unit: listings per month)	2019/1/1 - 2020/12/31
	Online reviews by guests	Inside Airbnb	Text content of guest reviews written in English. (Unit: text document)	2020/1/1 - 2020/12/31

All data used in this study is obtained from public sources that are open to Internet users. The only data that may contain identifiable personal information is Airbnb reviews, which were published out of Airbnb users’ own consent when they decided to post reviews online. Specifics of the identifiable personal information is not presented in the results or supplementary material of this paper. Furthermore, the collection and analysis of all data in this study fully comply with the terms and conditions for the source of the data. Consequently, as the study relied exclusively on publicly available and anonymized data, ethical review and approval from an Institutional Review Board and formal informed consent were waived.

*State of the pandemic* can be depicted by multiple indicators. This study chooses the most prevalent indicator, number of daily new COVID-19 positive cases in NYC, to represent the severity of the pandemic. Data related to COVID-19 in NYC is published daily by the NYC Department of Health and Mental Hygiene (NYC DOHMH), the government department responsible for NYC’s public health. Therefore, it’s considered official and reliable, and is used as a source for this research. The data from 2020/1/1 to 2020/12/31 is collected [46].

*Key events related to COVID-19* were identified and collected from a wide range of authoritative sources, including government bodies like the CDC, major news outlets such as The New York Times, and platform-specific announcements from Airbnb. This process ensured a comprehensive timeline of influential happenings from January 1, 2020, to December 31, 2020.

This study uses the data of Airbnb to analyze peer-to-peer accommodation. Airbnb is one of the earliest and the biggest platform in this industry, with its number of listings exceeding top 5 hotel brands combined, granting it sufficient representativeness. The data is acquired from Inside Airbnb (http://insideairbnb.com), a third-party website that collects, cleans, and provides public data gathered from Airbnb. Among academic literature about Airbnb, Inside Airbnb has been one of the most frequently adopted sources of data, enabling research to be conducted from word of mouth, pricing of housing, guests’ experience, and some other perspectives [47,48]. An updated collection of data is published every month, including price, location, introduction, reviews, and other information about listings from dozens of cities around the world.

*Online reviews left by the guests* for Airbnb listings are open information accessible to everyone. A tourist can write a review only after a booking takes place, so all the online reviews come from real bookings. Reviews’ text content, dates, and corresponding listings are included in the data that Inside Airbnb provides. The authors have acquired the reviews and their metadata of NYC Airbnb listings from January 2019 to December 2020.

*Supply of housing* is an important indicator of the market. Its volume may fluctuate along with multiple factors like hosts’ willingness, prosperity of the business, and interfere from the platform. If a host block a few dates on his/her calendar, this listing remains open for everyone to view, and the rest of dates can still be booked. It’s still available for data crawling by Inside Airbnb, thus still counts as an active listing in this research. If a host no longer want to rent out their home and closes the listing on Airbnb for good, data of this listing stops being viewed or collected by Inside Airbnb, therefore no longer exists in the monthly listing data. The supply in this study is defined as active listings that are still operated by their hosts. Each month’s data about Airbnb active listings in NYC from January 2019 to December 2020 is acquired. This paper examines the changes in booking volume by comparing the data in 2020–2019, also known as a year-over-year (YOY) rate.

*Room type* is an important feature of an Airbnb listing. There are four types: entire place, private rooms, shared rooms, hotel rooms. A guest of an entire place gets the whole space to themselves. A guest of a private room gets a whole bedroom but shares some public space with others. A guest of a shared room has to share both the bedroom and other space with others. A hotel room is more associated with traditional hotels. Since hotel rooms do not really belong to the category of peer-to-peer accommodation and are quite new and rare in NYC Airbnb market, this study only focuses on the other three types. Performance of each type as well as the sum volume is later examined to help understand travelers’ preference.

*Booking* is another important indicator of the market. As shown later in the paper, occupancy rate of listings was low during 2020, meaning bookings weren’t suppressed by lack of supply. Therefore, the volume of bookings basically represents the level of demand.

The data of bookings, however, is private information only accessible to guests, hosts, and Airbnb platform. Due to its unavailability, this research uses volume of online reviews as a proxy indicator. Not all guests write comments, so there are less online reviews than actual bookings. Still, the authors believe online review volume can represent the changes in bookings and demand of Airbnb market in this study, and the feasibility of adopting this indicator is justified in three folds. First, this paper uses a year-over-year (YOY) rate to examine the changes of booking volume, comparing data in 2020 with 2019. While bookings and reviews differ in their absolute value, their YOY shall by and large synchronize as long as the percentage of guests who write reviews among all guests stay stable, of which no opposite evidence has been shown. Second, several sources have shown that the percentage of guests who write reviews among all the guests are quite high, with the founder of Inside Airbnb concluding an eclectic review rate to be 50% [49]. Such a high percentage further validities online reviews’ representativeness of bookings. In addition, this proxy relation has already been adopted by multiple earlier research to calculate the revenue and influence of Airbnb [8,50]. Therefore, volume of online reviews shall be able to sufficiently reflect the fluctuation of Airbnb bookings.

Like the supply data, online reviews data here is also limited to the three main room types, eliminating ones that belong to hotel rooms.

### Data preprocessing and analysis

*State of the pandemic* is represented by number of daily new COVID-19 positive cases, which shows obvious fluctuation by a 7-day circle. This is likely due to the workweek system, where case report might get delayed and accumulated during the weekend and confirmed during weekdays. In order to eliminate this disturbance and better observe the trend of pandemic in NYC, a 7-day moving average value is calculated. The indicator of daily new COVID-19 positive cases in later context all refers to the 7-day moving average value of the original data.

*Key events related to COVID-19* are categorized and rated in several dimensions based on EST. Time-wise, occurrence date of the events are recorded. Space-wise, origins of the events are divided into four categories, which are global, national, state, and city. Strength-wise, each event is rated by the authors on each of the factor, namely novelty, disruption, and criticality. A three-point scale is used for rating, with 1 being the lowest strength and 3 being the highest. The scores of three factors are added up to be the overall strength of each event, ranging from 3 being the lowest and 9 being the highest. After screening by time, strength, and relevance with COVID-19 and peer-to-peer accommodation, nonsignificant and low-relevance events are dropped, and eventually 58 events were chosen are the key events for this study.

To analyze more accurately, the time scope of this research is divided into *3 phases* by key events as follows:

- The first wave: Feb. 26th - Jun. 7th. Milestones are the conformed possible community spread of COVID-19 in the U.S. and Vice President Pence being appointed to lead the federal government response to the COVID-19, both on Feb. 26th.
- Temporary recovery: Jun. 8th - Nov. 3rd. The milestone is the phase 1 of reopening in NYC on Jun. 8th.
- The second wave: Nov. 4th - Dec. 31st. The milestone is daily new positive cases climbing back to over 1000 per day on Nov. 3rd.

*Supply of housing* is examined by its YOY index. Since COVID-19 started spreading in the U.S. in early 2020 without affecting the market in 2019, YOY of 2020 over 2019 is a suitable approach to observe the influence of the pandemic on peer-to-peer accommodation market. A YOY index (number of listings in 2020 divided by that in 2019, then minus 1) of each month is calculated.

*Number of guests’ reviews, representing booking volume*, shows a pattern of fluctuation by a 7-day circle similar to daily new COVID-19 positive cases. This may result from workweek system’s influence on people’ traveling behavior, which isn’t relevant to the core of this research. Therefore, a 7-day moving average value is also applied for this indicator. Then, a daily YOY index (number of reviews in 2020 divided by that in 2019, then minus 1) is calculated based on the 7-day moving average value.

Therefore, volume of supply and reviews are analyzed in similar approaches, by examining their YOY index. Different room types are also examined. One difference here is that monthly data is used for supply, while reviews are detailed to the level of daily data.

*An STM model result is generated from the guests’ online reviews*. Python, Jupyter Notebook, and Rstudio are used during this procedure. First, the time of reviews is limited from 2020/1/1 to 2020/12/31, since the reviews in January and February of 2020 can be used as the data of before-COVID period and be compared to the reviews later in 2020. Since most of the reviews are written in English and this paper only conducts text analysis with English reviews, the languages used in the reviews are identified by cld2 language detector and non-English ones are then excluded. Reviews of hotel rooms are also ruled out. Eventually, 102157 English reviews for NYC Airbnb housing in 2020 remain as the raw data for the model building.

Next, using the STM package in RStudio, the text content of the reviews is read into the program and preprocessed by stemming, dropping punctuation, and removing stop words. Two covariates, date and room type of the review, are set. When deciding the number of topics for this model, the authors take into consideration earlier literature, where the number of topics ranges from 20 to 50. Several models are then tested using different number of topics to get the optimal combination of held-out likelihood and residuals, indicators of the preciseness of the model. Eventually, the model is set to contain 40 topics.

After the preprocessing is completed, a structural topic model consisting of 40 topics is generated from the prepared corpus and settings. The model itself doesn’t specify the core content of the topics, so instead of a definitive title, each topic is assigned with a number (1–40). However, words and reviews that are most closely related to each of the topic are generated by the program, therefore providing key information about subjects of the topics. After examining these words and reviews, the authors summed up the content and labelled all of the 40 topics with specific titles.

## Results

### Analysis of Airbnb booking volume

**Fig 4:**
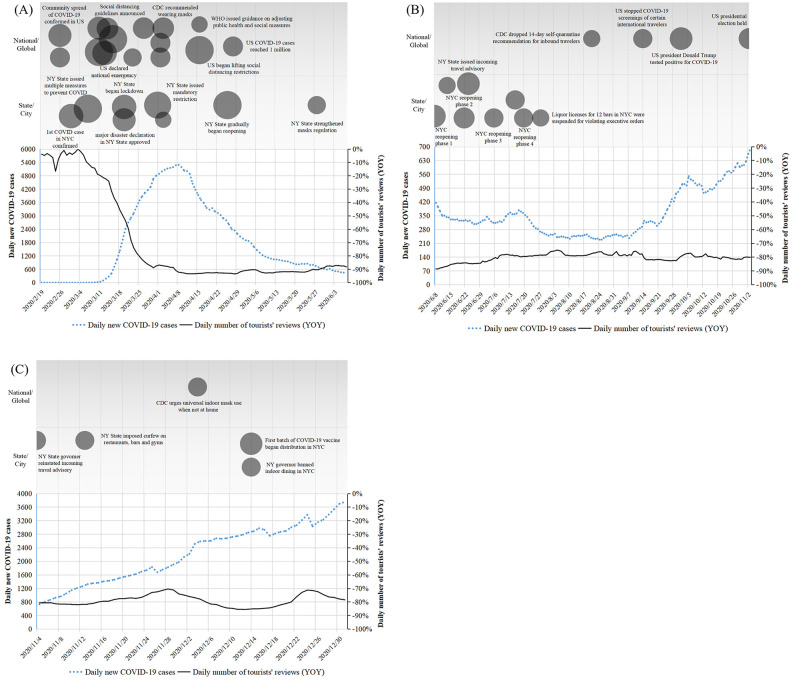
Exogenous events and YOY of tourists’ reviews during three phases.

Fig 4 shows the exogenous environment situation and YOY of Airbnb online reviews during the three phases. For each phase, number of daily new COVID-19 cases (representing state of the pandemic), relevant key events, and YOY of Airbnb online review volume (reflecting booking volume) are aligned together in one panel. Each bubble in the upper part of the Fig represents one event (or one event cluster), its area proportional to event’s overall strength. For the analysis and pictures to be clearer and more concise, some of the events which happened on proximate dates and are similar in essence are integrated into event cluster, and the strength of each cluster can top out at 12 to avoid weakening the significance of non-cluster events. Lower part of the Fig presents daily new COVID-19 case volume and review volume YOY. The three types of data share the same timeline on the bottom, enabling easy observation of the interaction among these factors.

Panel (A) of Fig 4 presents data during the first wave. COVID-19’s spreading in the U.S. was officially recognized at the end of February, and many events including various types of restrictions have taken place since then, shown by the gathering bubbles in March. Simultaneously, YOY of review volume has dropped drastically since March, once close to only −93%, indicating Airbnb bookings were severely damaged. Even though daily new cases and key events both began to ease from mid-April, Airbnb bookings remained extremely low during the first wave, with its YOY barely rose back to −90% until June.

Situation during the temporary recovery is presented in Panel (B) of Fig 4. Events are sparser and curve of daily COVID-19 cases is flatter compared to the first wave. State of the pandemic was remitting, NYC began to reopen, and restrictions are mostly lifted. Airbnb booking volume also gradually picked up during the summer, with its YOY above −80% in August. Since September, however, number of daily cases started to rise again, and the recovery of Airbnb booking came to a halt.

Panel (C) of Fig 4 presents the situation of the second wave. The number of daily new cases kept climbing up and the pandemic quickly worsened to a severe state, whereas few mandatory restrictions were issued this round, unlike the first wave. Airbnb reviews’ YOY shows some fluctuation but remained around −80% most of the time. It shows that bookings were less influenced by exogenous events than they were during the first wave.

As is shown in above panels, Airbnb booking volume dropped the most during the first wave, when exogenous events were strongest and most frequent. And it was most stable when key events were sparse, and number of daily new cases was constant. Therefore, **Proposition 1** is supported.

Events of various strength have exerted different influence on the market. Events such as conformation of the first case, declaring disaster emergency in the U.S. or NY State, and lockdown of NY state all possessed higher strength, and significantly affected the bookings volume. On the other hand, closing the playgrounds, curfew on NY bars & restaurants, and advisory about inter-state travel are some of the events that bear low strength, and Airbnb bookings were less affected by them. Therefore, **Proposition 3** is supported.

**Fig 5:**
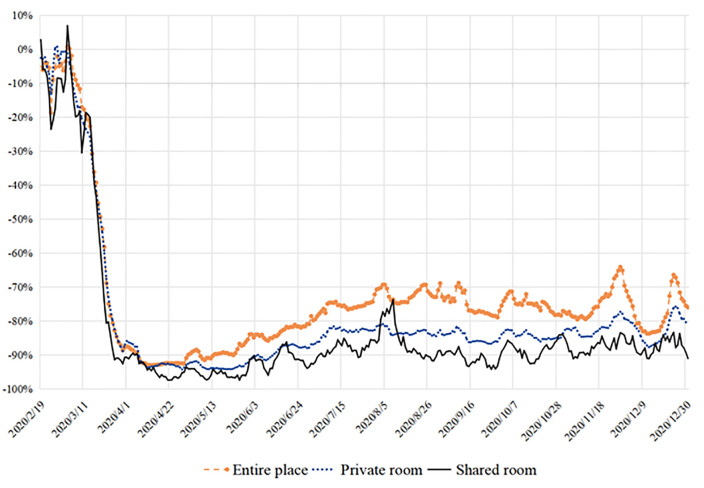
YOY of tourists’ reviews for different room types in three phases.

Fig 5 exhibits the YOY of online reviews of the three types of housing. From February to mid-March, bookings of the three types of housing all experienced apparent fluctuation. However, two differences among room types have become more obvious since April till end of the year. When it comes to the extent of overall decline, bookings of shared rooms dropped the most, while entire place dropped the least. In addition, fluctuation appears to be most wildly and frequent among shared room bookings, while the YOY of the other two types are gentler. Since an entire place provides the most social distance and a shared room provides the least, **Proposition 4a** is hereby supported.

### Analysis of Airbnb supply volume

**Fig 6:**
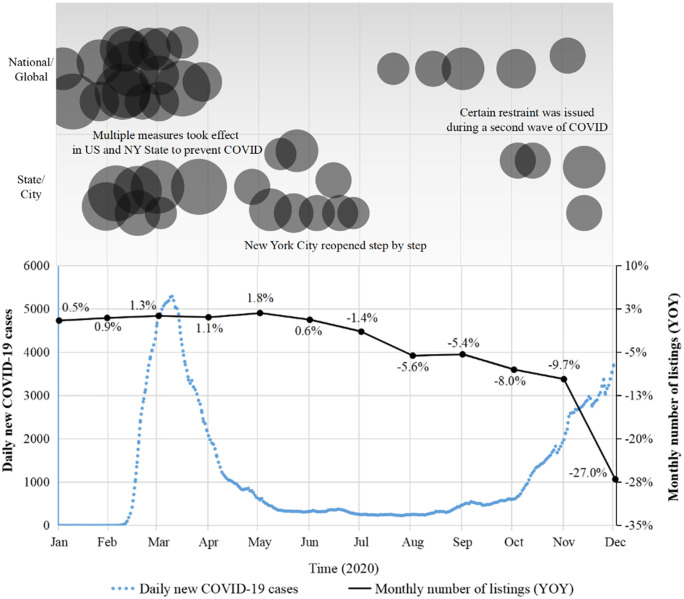
YOY of number of listings on Airbnb in New York city, 2020.

As shown in Fig 6, YOY of Airbnb listing volume in NYC kept steady from January to May. Since June, the index began to slowly decline by a small extent (less than 10%) until November. Eventually a sharp drop took place in December, lower the YOY to −27%.

The decline of housing supply mainly happened between June and December. However, as shown in the previous section on the Analysis of Airbnb booking volume, COVID-related exogenous events were strongest and most frequent from February to June, during which the housing supply didn’t exhibit obvious changes. Therefore, **Proposition 2** is not supported.

**Fig 7:**
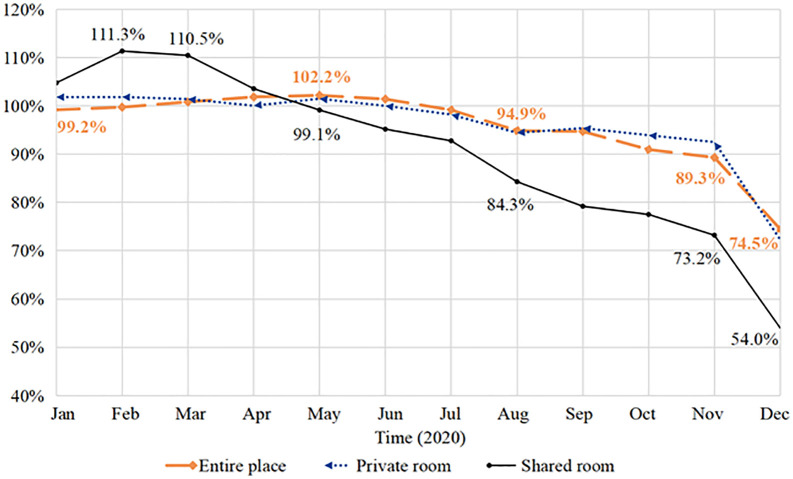
Listing volume YOY of different room types on Airbnb in NYC, 2020.

Fig 7 shows the housing supply YOY of different room types. Being the least isolated, shared rooms’ index began to decline as early as March and has plummeted most severely, dropping from 104.8% in January to 54% in December. The supply of entire places and private rooms didn’t start to drop till June, and their decrease is limited a smaller extent, with their YOY remaining above 70% till the end of 2020. In conclusion, **Proposition 4b** is supported.

### Analysis of Airbnb online reviews

A structural topic model with 40 topics is generated from the corpus of NYC Airbnb online reviews in 2020. Plenty information is covered, including location, transportation, facilities, service from hosts, etc. The authors labelled all the topics by their core content. Table 2 shows the 40 topics and each topic’s proportion in the model.

**Table 2:** Structural topic model of NYC Airbnb online reviews in 2020.

Number	Label	Proportion	Number	Label	Proportion
1	Beach and transportation alongside	0.9%	21	Sanitary	1.7%
2	Lodging in downtown Manhattan	1.0%	22	Convenience of the lodging’s location	7.3%
3	Host offering information and guide	1.5%	23	Response time of the host	3.8%
4	Family travel	1.3%	24	Booking the lodging	2.1%
5	Praising hosts and willing to revisit	8.4%	25	Good experience and willing to recommend	3.7%
6	Public transportation	3.1%	26	Check-in and check-out	1.4%
7	Complimenting space of the lodging	2.7%	27	Solo travel	0.7%
8	Hosts’ communication and check-in	2.9%	28	Bed and its comfortableness	2.1%
9	Noise and cancelling reservation	3.4%	29	Lodging being a good deal	7.3%
10	COVID-19	1.1%	30	Feeling at home	2.6%
11	Willingness to revisit	3.0%	31	Lodging matching the pictures	1.9%
12	Complimenting lodging experiences	3.0%	32	Shared space	2.2%
13	Comfortable space	2.8%	33	Dissatisfied with lodging experiences	2.8%
14	Nice view and environment	1.5%	34	Kitchen and food	1.8%
15	Exceeding expectation	1.7%	35	Lodging meeting guests’ needs	2.0%
16	Outdoor recreational area	2.1%	36	Items of dissatisfaction	0.6%
17	Complimenting the host	0.9%	37	Best lodging experience	1.6%
18	Traveling in NYC	1.4%	38	Close to commercial district	4.8%
19	Praising and recommending the lodging	2.1%	39	Animals and pets	1.1%
20	Lodging in Brooklyn	2.8%	40	Good lodging in NYC	1.1%

After viewing and identifying all of the 40 topics, three topics relevant to COVID-19 are picked out and analyzed. Topic 10, labelled COVID-19, most directly represents guests’ thoughts about the pandemic. Topic 21 (sanitary) and topic 32 (shared space) are also assumed to grow in prevalence since people likely emphasize hygiene and social distance more after the pandemic took place. Proportions of these three topics are to be examined to verify if Proposition 5 and Proposition 6 can be supported.

In this segment, the details of each topic, including its top words and overall proportion, are first exhibited. Then the paper examines how the prevalence of the topic (represented by topic proportion) changes with time, accompanied by the fluctuating state of the pandemic and Airbnb booking volume. Ups and falls of topic prevalence in different room types are also inspected, but limited to entire place and private room, since the volume of shared rooms is too small for STM to generate its very topic proportion.

Topic 10 is labeled COVID-19, topic 21 is labeled Sanitary, and topic 32 is labeled Shared Space. Details of these three topics are presented in Table 3, including representative top words within the topics and the proportion of each topic in the model.

**Table 3:** Details of Topic 10, Topics 21, and Topic 32.

Number	Label	Method of Word-topic Association	Top Words	Proportion
10	COVID-19	Highest Probability	work, month, due, end, covid, situation, extend, pandemic, done, renovate	1.1%
		FREX	coronavirus, covid, -term, crisis, month, pandemic, covid-, gym, jason	
		Lift	-floor, bait, cdc, contrast, cookbook, dab, dena’, frontline, gatsby, hobby	
		Score	month, due, covid, pandemic, end, done, covid-, job, situation	
21	Sanitary	Highest Probability	shower, water, bed, towel, smell, hot, bathroom, like, also, floor	1.7%
		FREX	paper, pressure, sheet, wash, toilet, body, hair, stain, soap, cloth	
		Lift	baseboard, basin, bruise, cage, clue, corn, crevice, crusty, drape, earring	
		Score	shower, dirty, bed, towel, toilet, water, sheet, hot, smell, paper	
32	Shared Space	Highest Probability	room, bathroom, live, bedroom, private, small, share, guest, entrance, common	2.2%
		FREX	room, staff, desk, separate, share, roommate, common, private, hostel, master	
		Lift	daniela, flex, furthest, hairdress, mabel, wider, groom, westhouse, da’, l-train	
		Score	room, bathroom, bedroom, live, private, share, small, desk, common, staff	

**Fig 8:**
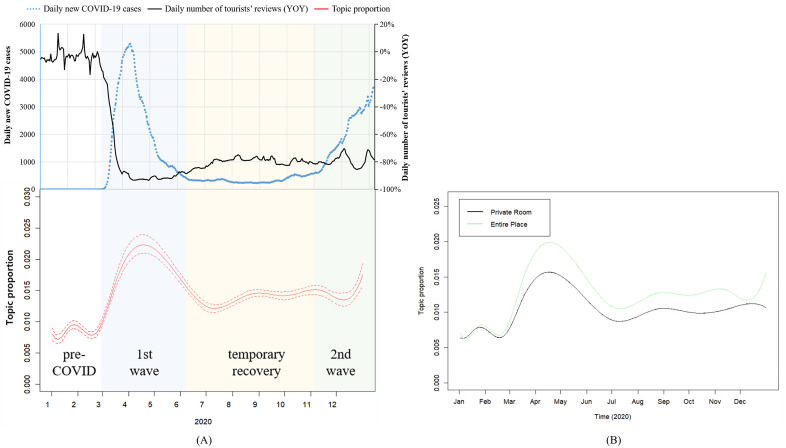
Trend of topic 10 (COVID-19), 2020.

Trends of topic 10 (COVID-19), along with exogeneous environment, is presented in Fig 8. Panel (A) shows the proportion of topic COVID-19, with dashed lines representing 95% confidence intervals. The fluctuation of topic COVID-19’s proportion highly coincides with the state of the pandemic, and reversely coincides with tourists’ reviews volume (YOY). The raise in daily new COVID-19 cases is often followed by higher topic proportion and decreased Airbnb booking.

Panel (B) shows the prevalence of topic COVID-19 by different room type. Two lines share a similar trend, but the proportion is higher in entire place almost all the time than in private room.

**Fig 9:**
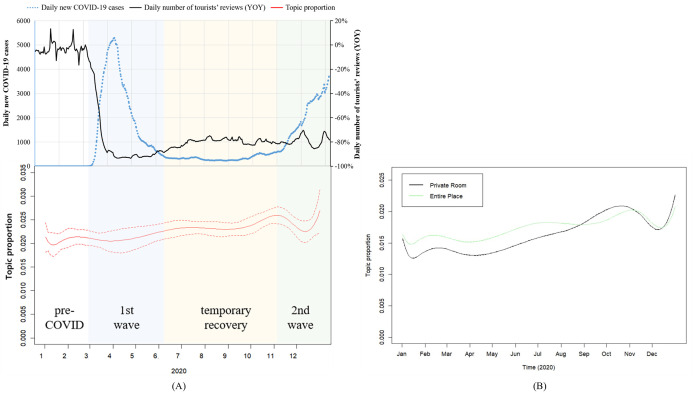
Trend of Topic 21 (Sanitary), 2020.

Trends of topic 21 (Sanitary) and environment factors are shown in Fig 9. The proportion of topic sanitary shows less synchronization with the severity of the pandemic compared to topic COVID-19, with dashed lines in panel (A) representing 95% confidence intervals. But overall, its topic prevalence still gradually rises across the whole year. As shown in panel (B), as time went by, guests of private room raised their emphasis on this subject by a larger extent than those of entire place.

**Fig 10:**
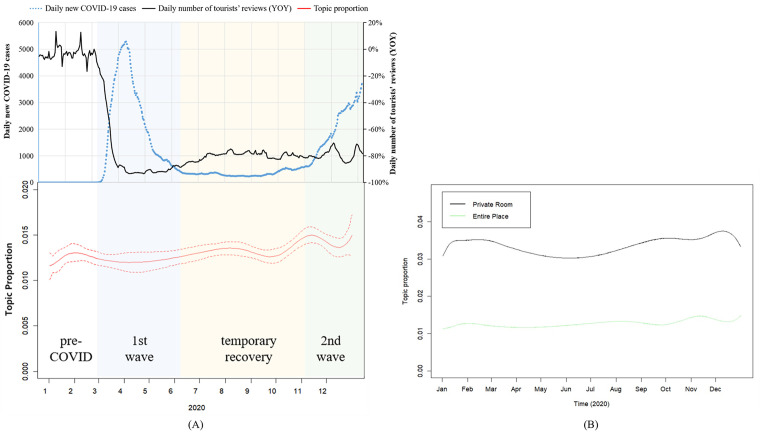
Trend of topic 32 (Shared Space), 2020.

Trends of topic 32 (Shared Space) and environment factors are shown in Fig 10. The prevalence curve of topic Shared Space shares a similar trend with topic Sanitary), with dashed lines in panel (A) representing 95% confidence intervals. A general growth throughout the year is observed, but monthly synchronization with pandemic situation or Airbnb booking isn’t significant. Guests of private rooms paid larger attention to this subject than guests of entire places, but change of topic prevalence hasn’t shown significant difference between two room types.

Generally speaking, after COVID-19 hit, guests paid more attention to the topics relevant to the pandemic. Though the synchronization isn’t so quick during a short time period, the broader trends of the topic prevalence in the long run still rise along with the intensification of the pandemic. **Proposition 5** is supported.

Among different room types, guests of private rooms have shown more obvious change of attention toward the topics of sanitary and shared space than guests of entire place. However, attention to the topic COVID-19 is changed more obviously among guests of entire place. Overall, **Proposition 6** is still supported, but to a relatively weak extent.

Testing results of P1-P6 is shown in Table 4.

**Table 4:** Results of Proposition Testing (P1-P6).

Proposition		Result
P1	During the pandemic, when COVID-19-related key events are most numerous, most frequent, and strongest, peer-to-peer accommodation booking volume undergoes the highest level of decline.	Supported
P2	During the pandemic, when COVID-19-related key events are most numerous, most frequent, and strongest, peer-to-peer accommodation supply volume undergoes the highest level of decline.	Not supported
P3	During the pandemic, strength of COVID-19-related key events has a negative influence on peer-to-peer accommodation bookings volume.	Supported
P4a	During the pandemic, less isolated listings, where social distant are hard to maintain, experience larger degree of changes in booking volume.	Supported
P4b	During the pandemic, less isolated listings, where social distant are hard to maintain, experience larger extent of changes in supply volume.	Supported
P5	After COVID-19 hit, guests of peer-to-peer accommodation pay more attention to sanitary condition, social distance, and other COVID-19-related issues than pre-pandemic time. The rise of these attention synchronizes with development of the pandemic.	Supported
P6	During the pandemic, the extent of changes in guests’ attention to sanitary condition, social distance and other COVID-19-related issues vary among different types of listings. Larger extent of changes in attention is found among guests of less isolated listings where social distance is harder to maintain.	Supported

**Fig 11:**
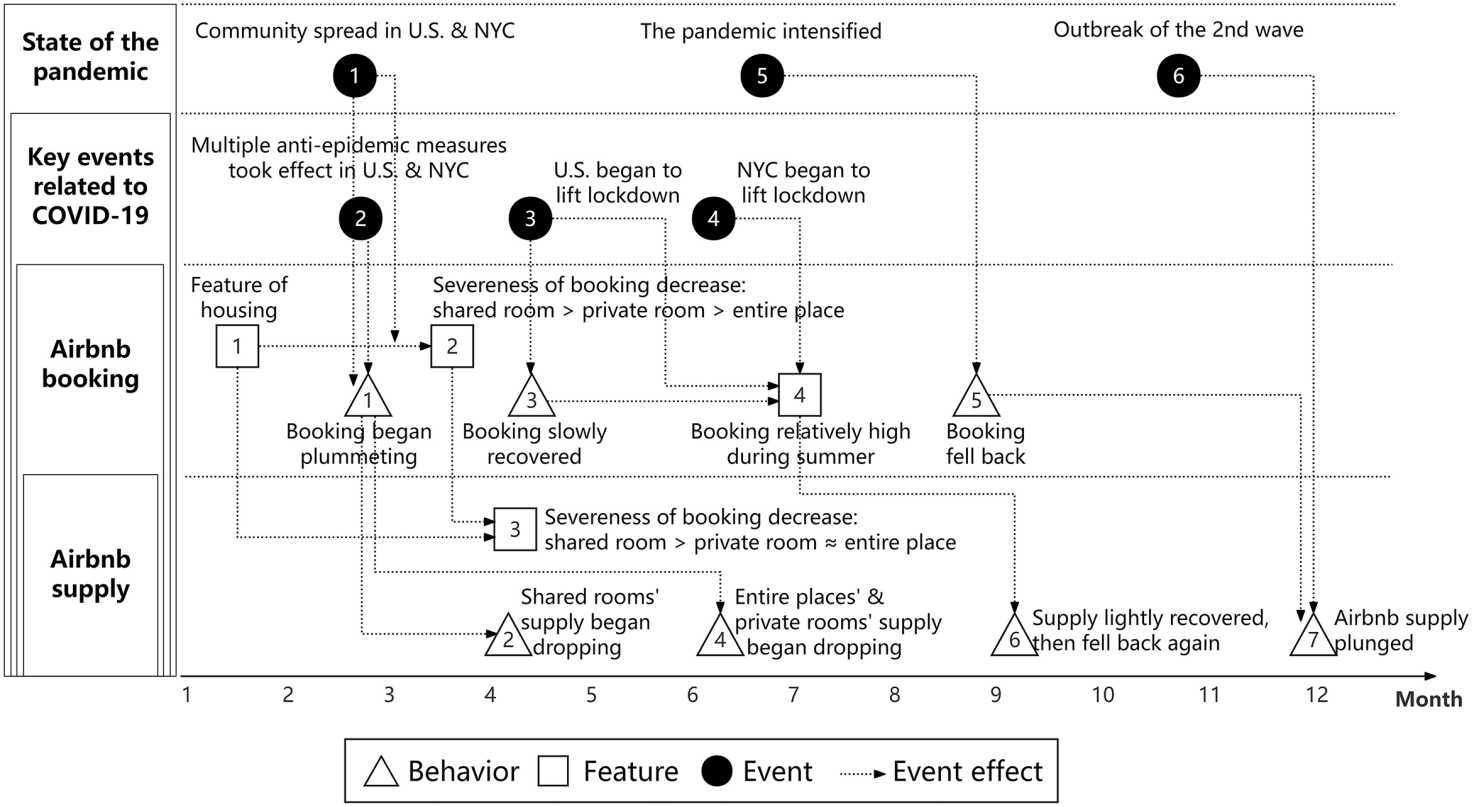
Exogenous events and corresponding effects on NYC Airbnb in 2020.

Using the prototype of EST, the significant events, behaviors and features regarding COVID-19 and NYC Airbnb in 2020 are integrated into a diagram (Fig 11) to visualize the interaction among these interrelated elements. Fig 11 shows that the spread of the COVID-19 and related measures lead to guests reducing their staying in Airbnb and developing preference about room types. Then the plummet in booking volume, along with further worsening of the pandemic, resulted in the decline of housing supply. As the state of pandemic and booking amount both have moved upward and downward, Airbnb supply also went through fluctuation in both directions. In addition, though not included in this Fig, online reviews show that guests’ attention towards COVID-19 also began to boost since early March, about the same time that booking volume began plummeting. Therefore, it’s plausible to conclude that exogenous events raised people’s concern, which further caused the plunge of Airbnb booking.

## Discussion

### Discussion of findings

Findings of this study confirm COVID-19’s enormous damage to home-sharing market, including both demand and supply end. On top of that, more comprehensive insights about the nuanced elaborate dynamics of various factors are also provided.

*Tourists’ foci* *related to COVID-19 shift simultaneously with severity of pandemic, related key events, and Airbnb booking volume*. The closer related to the pandemic, more concurrent the fluctuation appeared to be. Therefore, the causality between the pandemic and market performance is solidified by tourists exhibiting their considerations. Larger extent of change in attention and booking is found in less isolated housing, which further strengthens the connection.

*Different patterns of fluctuation are observed between demand and supply.* Bookings reacted immediately and sharply to external events, plunging during the first wave in synchronization with public health alerts. In contrast, the supply of listings showed significant inertia, with a much more delayed and less severe decline that only accelerated late in the year. This lag suggests a fundamental difference in decision-making: guests’ choices are immediate and driven by health risk perception, while hosts’ decisions are more strategic and economically calculated, involving sunk costs and long-term viability. Hosts likely adopted a ‘wait-and-see’ approach, only exiting the market after a sustained period of depressed demand convinced them the crisis would be prolonged.

*Stronger and more frequent events incite more drastic changes to the market*. Many policies issued by the government were targeted to ease the spread of COVID-19, but strength of these regulations varies widely. Some are strictly forced or far-reaching, while some are mere guidance, advisory, or small-ranged. Their influences on the market turn out to be very different. The succession of lockdown and other mandatory orders significantly lowered Airbnb booking in March, while the scattering restrictions on merely a few industries during the second wave didn’t generate as much influence.

*Timing affects the influence of exogenous events upon peer-to-peer accommodation market*. The two waves of pandemic in NYC in 2020 were both very severe. During the first wave, Airbnb bookings suffered critically, while supply remained almost unchanged. Amid the second wave, however, bookings volume was stable, while supply showed significant decline. Two event clusters, similar in many aspects but distinct in timing, eventually caused very different effects on the same entity. Many factors may have played their parts here. First, novelty of the event reduces when it happens a second time. Meanwhile, the entity may have updated their coping pattern after prior experience and adaptation. Attitude-wise, participants may get tired and lessen their reaction later in the process (like the booking volume), or on the contrary they may experience an overlapping of precedent and current influence and react even more strongly (like the supply volume). All these factors combined can act on similar events and generate different results.

### Theoretical implications

This study offers several key theoretical contributions to both the peer-to-peer (P2P) accommodation literature and Event System Theory (EST). Its primary contribution lies in identifying and empirically demonstrating an asynchronous response mechanism between guests and hosts in the face of a systemic crisis. While prior literature confirmed that crises damage the sharing economy, it did not fully explain the nuanced temporal dynamics of risk decisions among different platform participants. Our findings reveal a specific causal pathway: external events first trigger an immediate, perception-driven risk-aversion response from guests (demand side), reflected in plummeting bookings and a simultaneous shift in expressed concerns (e.g., hygiene). Only after a sustained period of depressed demand does this translate into a delayed, economically-driven risk-mitigation response from hosts (supply side), who eventually delist their properties. This uncovers a critical pattern in the triadic dynamic of event–guest–host, where the guest acts as a rapid-reacting intermediary translating external shocks into direct market pressure on the host.

Second, this research substantiates the subtle economics of the sharing economy by specifying how different risk sensitivities shape market behavior. We move beyond general observations by demonstrating that: Demand reacts faster and more sharply than supply because guests’ risk decisions (canceling a trip) are immediate and driven by health-peril perception, whereas hosts’ decisions (delisting a property) are strategic, involving calculations of sunk costs and long-term financial viability. Risk perception directly translates into strategic preference, as shown by the clear shift toward more isolated listings (entire places). This provides concrete evidence of how consumers actively manage risk by altering their consumption choices within the platform’s offerings. These findings specify the behavioral mechanisms underpinning market elasticity in the P2P context, showing how interdependent but distinct risk-taking calculations by guests and hosts collectively shape market outcomes during a crisis.

Finally, we enrich EST in its construct, coverage, and presentation for hospitality and risk management contexts. In terms of construct, we operationalize event strength not abstractly but through a multi-dimensional rating and link it empirically to both behavioral and perceptual metrics. Regarding coverage, we extend EST’s application to platform-based, non-institutional settings, demonstrating how consumers’ risk-taking decisions are shaped by event sequences and property features. For presentation, the timeline-based diagrams are not merely illustrative; they formally visualize the proposed sequential mechanism and the lagged host–guest responses, clarifying temporal relationships that are often assumed but rarely demonstrated in event-oriented research.

### Managerial implications

*The findings provide hospitality practitioners with reference for future risk management*. Conclusions about the different response of demand and supply, as well as the subtle impact of timing, help business participants better understand the law of consumers’ reaction, adjust their strategies, and minimize their loss during crisis coping.

*Insights about events’ manifold influence on the industry help governments optimize policies and regulation, especially when dealing* *with public emergencies and health events.* Depending on external environment and specific targets, governments can select from multiple categories of approaches, which vary in their novelty, disruption, and criticality. More suitable and effective policies can be formulated to induce the desired reaction from the community and generate a promising result.

### Limitations and directions for future research

The time range of this study is within the year of 2020. Though its representative, delving into the data of later years can bring new insights into the investigation. Future studies may include a later time range, especially with the development of vaccine and other updates, to reach a more comprehensive view of the whole picture.

Data of this study is spatially limited to New York City. While being a valid choice of region, this alone doesn’t embody the diversity of the peer-to-peer accommodation worldwide. Exploration into other regions of different economic environment and cultural background can enrich the insight of interaction among the business participants.

Volume of reviews is used as a proxy indicator of volume of bookings. If future study can acquire actual bookings volume or data from other home-sharing platform, the elaborate dynamics among the guest, the host, and the post can be examined and enhanced.

## Conclusions

This paper investigates the peer-to-peer accommodation industry under the influence of COVID-19 pandemic. Facing the unprecedented health risk, Airbnb guests quickly reduced their behavior of traveling and outside-lodging, at the similar pace of showing their concerns about the pandemic, sanitary, and social distancing issues in their online reviews. Hosts of accommodation also reduced the housing supply, although the pattern of decrease is different from the guests. The whole industry suffered drastic loss, especially during the period filled with strong and frequent exogenous events. Meanwhile, the impact of events’ timing is more subtle. The findings combine market statistics and guests’ online reviews, therefore construct a solid chain from outside events, to consumers’ mindset, and to consumers’ behavior in this comprehensive setting. This composition of study introduces a more integral approach to explore customers’ thoughts and behavior. Based on fine-grained data, the research dug into details and investigates the different modes of behavior from both demand side and supply side, adding to the insights of intertwined market movement during public crises. The research enriches EST, and provides government and hospitality practitioners with reference for future risk management.

## Supporting information

S1 File Supplementary Materials. (ZIP)

## References

[1] World Health Organization. Coronavirus disease (COVID-19). https://www.who.int/news-room/fact-sheets/detail/coronavirus-disease-(covid-19). Accessed 2024 August 31.

[2] World Bank. World development report 2022: Finance for an equitable recovery. The World Bank. 2022.

[3] Craft 4. Airbnb vs. the global hotel industry. 2019.

[4] Airbnb Inc. Airbnb, Inc. 2020 annual report 10-K. 2021.

[5] Kowalczyk-Anioł J, Kacprzak K, Szafrańska E. How the COVID-19 Pandemic Affected the Functioning of Tourist Short-Term Rental Platforms (Airbnb and Vrbo) in Polish Cities. Int J Environ Res Public Health. 2022;19(14):8730. doi: 10.3390/ijerph19148730 35886586 PMC9321625

[6] Milone FL, Gunter U, Zekan B. The pricing of European airbnb listings during the pandemic: A difference-in-differences approach employing COVID-19 response strategies as a continuous treatment. Tour Manag. 2023;97:104738. doi: 10.1016/j.tourman.2023.104738 36777288 PMC9899787

[7] Liang S, Leng H, Yuan Q, Yuan C. Impact of the COVID-19 pandemic: Insights from vacation rentals in twelve mega cities. Sustain Cities Soc. 2021;74:103121. doi: 10.1016/j.scs.2021.103121 34540564 PMC8437680

[8] Chen G, Cheng M, Edwards D, Xu L. COVID-19 pandemic exposes the vulnerability of the sharing economy: a novel accounting framework. Platform-Mediated Tourism. Routledge. 2022:213–30. doi: 10.4324/9781003230618-12

[9] Abouk R, Heydari B. The immediate effect of COVID-19 policies on social distancing behavior in the United States. medRxiv. 2020. doi: 10.1101/2020.04.07.20057356 PMC8093844 33400622

[10] Hu F, Pan J, Wang H. Unveiling the spatial and temporal variation of customer sentiment in hotel experiences: a case study of Beppu City, Japan. Humanit Soc Sci Commun. 2024;11(1). doi: 10.1057/s41599-024-04226-4

[11] Yu J, Seo J, Hyun SS. Perceived hygiene attributes in the hotel industry: customer retention amid the COVID-19 crisis. Int J Hosp Manag. 2021;93:102768. doi: 10.1016/j.ijhm.2020.102768 36919179 PMC9998174

[12] Kim H, Li J, So KKF. Enhancing Consumer Confidence and Response Efficacy in Tourism: Typology and Effectiveness of the Hotel Industry’s Responses to COVID-19. J Travel Res. 2023;62(4):907–25. doi: 10.1177/00472875221095211 36883176 PMC9978237

[13] Ju Y, Jang SS. The Effect of COVID-19 on hotel booking intentions: Investigating the roles of message appeal type and brand loyalty. Int J Hosp Manag. 2023;108:103357. doi: 10.1016/j.ijhm.2022.103357 36246515 PMC9537292

[14] Benítez-Aurioles B. How the peer-to-peer market for tourist accommodation has responded to COVID-19. IJTC. 2021;8(2):379–92. doi: 10.1108/ijtc-07-2021-0140

[15] Cheng M, Hu M, Lee A. A global perspective on the impact of COVID-19 on peer-to-peer accommodation: human mobility, case number and lockdown policies. IJCHM. 2023;35(8):2838–67. doi: 10.1108/ijchm-02-2022-0221

[16] Wang N, Hincks S. Airbnb and the COVID-19 pandemic: a geospatial analysis of Greater London. Environment and Planning B-Urban Analytics and City Science. 2024. doi: 10.1177/23998083241302557

[17] Jang S, Kim J, Kim J, Kim S (Sam). Spatial and experimental analysis of peer-to-peer accommodation consumption during COVID-19. Journal of Destination Marketing & Management. 2021;20:100563. doi: 10.1016/j.jdmm.2021.100563

[18] Nicolau JL, Sharma A, Shin H, Kang J. Airbnb vs hotel? Customer selection behaviors in upward and downward COVID-19 trends. IJCHM. 2023;35(12):4384–406. doi: 10.1108/ijchm-04-2022-0478

[19] Filieri R, Milone FL, Paolucci E, Raguseo E. A big data analysis of COVID-19 impacts on Airbnbs’ bookings behavior applying construal level and signaling theories. Int J Hosp Manag. 2023;111:103461. doi: 10.1016/j.ijhm.2023.103461 36998942 PMC9998299

[20] Ye S, Lei SI, Zhao X, Zhu L, Law R. Modeling tourists’ preference between hotels and peer-to-peer (P2P) sharing accommodation: a pre- and post-COVID-19 comparison. International Journal of Contemporary Hospitality Management. 2023;35(4):1423–47. doi: 10.1108/IJCHM-12-2021-1556

[21] Zhong L, Liu J, Morrison AM, Dong Y, Zhu M, Li L. Perceived differences in peer-to-peer accommodation before and after COVID-19: evidence from China. IJCHM. 2023;35(4):1539–61. doi: 10.1108/ijchm-12-2021-1557

[22] Liu Q, Mengoni P. What do Airbnb users care about before, during and after the COVID-19? An analysis of online reviews. In 2023 IEEE International Conference on Web Intelligence and Intelligent Agent Technology (WI-IAT): 2023.

[23] Buzzacchi L, Milone FL, Paolucci E, Raguseo E. How to react to a shock? Effects of Airbnb hosts’ choices and market segmentation at the time of Covid-19. Information & Management. 2023;60(7):103857. doi: 10.1016/j.im.2023.103857

[24] Boto-García D. Heterogeneous price adjustments among Airbnb hosts amid COVID-19: Evidence from Barcelona. Int J Hosp Manag. 2022;102:103169. doi: 10.1016/j.ijhm.2022.103169 35095167 PMC8784670

[25] Farmaki A, Miguel C, Drotarova MH, Aleksić A, Časni AČ, Efthymiadou F. Impacts of Covid-19 on peer-to-peer accommodation platforms: Host perceptions and responses. Int J Hosp Manag. 2020;91:102663. doi: 10.1016/j.ijhm.2020.102663 32901166 PMC7470711

[26] Peng C, Peng Z, Lin J, Xie J, Liang Y. How and when perceived COVID-19 crisis disruption triggers employee work withdrawal behavior: The role of perceived control and trait optimism. Personality and Individual Differences. 2025;235:112981. doi: 10.1016/j.paid.2024.112981

[27] Morgeson FP, Mitchell TR, Liu D. Event System Theory: An Event-Oriented Approach to the Organizational Sciences. AMR. 2015;40(4):515–37. doi: 10.5465/amr.2012.0099

[28] He J, Zhang Y. Urban epidemic governance: An event system analysis of the outbreak and control of COVID-19 in Wuhan, China. Urban Stud. 2023;60(9):1707–29. doi: 10.1177/00420980211064136 37416838 PMC10311380

[29] Liu S, Liu Y, Guo M, Wang R, Sun Q, Zhu R. Formation mechanism and governance strategies of stigma in public health emergencies: Based on event system theory. Front Public Health. 2023;10:1067693. doi: 10.3389/fpubh.2022.1067693 36711340 PMC9874323

[30] Wong WM, Su W. COVID-19 effect on retired lifestyle intention: The two-order confirmatory factory analysis in the structural equation model. Linguistica Antverpiensia. 2020;2020:58–75.

[31] Li X, Song Y, Hu B, Chen Y, Cui P, Liang Y, et al. The effects of COVID-19 event strength on job burnout among primary medical staff. BMC Health Serv Res. 2023;23(1):1212. doi: 10.1186/s12913-023-10209-z 37932737 PMC10629111

[32] Liu Y, Zhang Z, Zhao H, Liu L. The influence of the COVID-19 pandemic on work connectivity behavior. Front Psychol. 2023;14:831862. doi: 10.3389/fpsyg.2023.831862 36844306 PMC9947784

[33] Liu Y, Zhang Z, Zhao H. The Influence of the COVID-19 Event on Deviant Workplace Behavior Taking Tianjin, Beijing and Hebei as an Example. Int J Environ Res Public Health. 2020;18(1):59. doi: 10.3390/ijerph18010059 33374789 PMC7794894

[34] Zhang J, Zheng W, Hua W, Fu M. Making sense of the impact of COVID-19 event on college students’ online deviant behavior: does stress-is-enhancing mindset matter? Curr Psychol. 2023;43(14):12495–507. doi: 10.1007/s12144-023-05361-y

[35] McFarland LA, Reeves S, Porr WB, Ployhart RE. Impact of the COVID-19 pandemic on job search behavior: An event transition perspective. J Appl Psychol. 2020;105(11):1207–17. doi: 10.1037/apl0000782 33030925

[36] Bail C. Topic modeling. https://cbail.github.io/SICSS_Topic_Modeling.html. Accessed 2023.

[37] Roberts ME, Stewart BM, Tingley D, Airoldi EM. The structural topic model and applied social science. In: Advances in neural information processing systems workshop on topic models: computation, application, and evaluation; Harrahs and Harveys, Lake Tahoe, 2013.

[38] Roberts ME, Stewart BM, Tingley D. stm: An R Package for Structural Topic Models. J Stat Soft. 2019;91(2). doi: 10.18637/jss.v091.i02

[39] Gao B, Zhu M, Liu S, Jiang M. Different voices between Airbnb and hotel customers: An integrated analysis of online reviews using structural topic model. Journal of Hospitality and Tourism Management. 2022;51:119–31. doi: 10.1016/j.jhtm.2022.03.004

[40] Abramova O, Batzel K, Modesti D. Collective response to the health crisis among German Twitter users: A structural topic modeling approach. International Journal of Information Management Data Insights. 2022;2(2):100126. doi: 10.1016/j.jjimei.2022.100126

[41] Jo W, Lee J, Park J, Kim Y. Online Information Exchange and Anxiety Spread in the Early Stage of the Novel Coronavirus (COVID-19) Outbreak in South Korea: Structural Topic Model and Network Analysis. J Med Internet Res. 2020;22(6):e19455. doi: 10.2196/19455 32463367 PMC7268668

[42] Lu J, Liu J. Communicating concerns, emotional expressions, and disparities on ethnic communities on social media during the COVID-19 pandemic: A structural topic modeling approach. American Behavioral Scientist. 2023. doi: 10.1177/00027642231164046

[43] Lee KR, Kim B, Nan D, Kim JH. Structural Topic Model Analysis of Mask-Wearing Issue Using International News Big Data. Int J Environ Res Public Health. 2021;18(12):6432. doi: 10.3390/ijerph18126432 34198600 PMC8296260

[44] Wang Y. Emergency risk communication: A structural topic modelling analysis of the UK government’s COVID-19 press briefings. Nordic Journal of English Studies. 2022;21(2):226–51. doi: 10.35360/njes.782

[45] Company N. NYC & Company Annual Report 2020. 2021.

[46] NYC Department of Health and Mental Hygiene. https://www.nyc.gov/site/doh/index.page. 2021. Accessed 2021.

[47] Sutherland I, Kiatkawsin K. Determinants of Guest Experience in Airbnb: A Topic Modeling Approach Using LDA. Sustainability. 2020;12(8):3402. doi: 10.3390/su12083402

[48] Grant R, Zu H, Roy SS. Unexpected urban comparisons: Airbnb-ization in Cape Town, South Africa, and Broward County’s coastal cities, Florida, United States. Cities. 2024;155:105455. doi: 10.1016/j.cities.2024.105455

[49] Cox M. Inside Airbnb 2022. http://insideairbnb.com/data-assumptions. Accessed 2023 October 1.

[50] Simcock T, Smith D. The bedroom boom: Airbnb and London. 2016.

