# Supplementary material for: Decisions about risk taking: Elaborate dynamics between guests and hosts of peer-to-peer accommodation during COVID-19
Source: PLoS One. 2026 Mar 16;21(3):e0341733. doi: 10.1371/journal.pone.0341733 (PMC12991232; doi:10.1371/journal.pone.0341733)
Supplement: S1 File — (ZIP) [file pone.0341733.s001.zip › Codes - Data Processing and Analyzing (in RStudio).docx]

---

title: "R Notebook"

output:

pdf_document: default

html_document:

df_print: paged

---

# 0. install package

```{r}

library(NLP)

library(stm)

library(tm)

library(tidyr)

library(stringr)

library(tidytext)

library(plyr)

library(tidyverse)

library(RColorBrewer)

library(igraph)

library(Rtsne)

library(rsvd)

library(geometry)

library(cld2)

library(wordcloud)

library(data.table)

library(readxl)

```

# 1. Official Processing

## 1.1 Ingest (read & identify language & textProcessor & prepDocuments?)

```{r}

# Read using xlsx

data_ny_original <- read_excel("reviews_2020_original.xlsx", na = "", )

data_header <- read.csv(file = "header.csv",encoding = 'UTF-8')

data_ny <- rbind(data_header, data_ny_original)

data_ny <- data_ny[-1,]

# Filtering by Languages

data_ny$lang = detect_language(data_ny$reviews)

data_ny_en = subset(data_ny, lang == "en")

data_ny_en_entire = subset(data_ny_en, room_type == "Entire home/apt")

data_ny_en_private = subset(data_ny_en, room_type == "Private room")

data_ny_en_shared = subset(data_ny_en, room_type == "Shared room")

rm(data_ny_en_private)

data_ny_en = data_ny_en[-10]

# write.csv(data_ny, file = 'reviews language tag.csv', sep = ",", quote = TRUE)

detect_language_mixed(data_ny_original$reviews)

```

```{r}

save.image("D:/Analysis_0303.RData")

```

```{r}

custom_stopwords <- read.csv("stopwords_clean.csv", header = FALSE)

vector_custom_stopwords <- as.vector(custom_stopwords)

processed_ny_en <- textProcessor(data_ny_en$reviews, metadata = data_ny_en,

customstopwords = vector_custom_stopwords$V1,

language = "en", )

```

## 1.2 Prepare (plotRemoved & prepDocuments)

```{r}

plotRemoved(processed_ny_en$documents, lower.thresh = seq(0, 100, by = 1))

out <- prepDocuments(processed_ny_en$documents,

processed_ny_en$vocab,

processed_ny_en$meta,

lower.thresh = 2)

thoughts_text <- data_ny_en$reviews[-processed_ny_en$docs.removed]

thoughts_text <- thoughts_text[-out$docs.removed]

length(thoughts_text)

```

## 1.3 Estimate (heldout & stm)

```{r}

heldout <- make.heldout(out$documents, out$vocab)

eval.heldout(model_k40, heldout$missing)

```

```{r}

model_k40 <- stm(documents = out$documents, vocab = out$vocab, K = 40,

prevalence =~ room_type + s(date_number) + room_type * s(date_number),

max.em.its = 75,

data = out$meta, init.type = "Spectral")

```

## 1.4 Evaluate (selectModel & searchK)

```{r}

storage <- searchK(out$documents, out$vocab, K = c(20, 22, 24, 26, 28, 30, 32, 34, 35, 38, 40,

42, 44, 46, 48, 50, 52, 54, 56, 58, 60),

init.type = 'Spectral',

prevalence =~ room_type + s(date_number) + room_type * s(date_number),

data = out$meta,

max.em.its = 3)

storage_less <- searchK(out$documents, out$vocab, K = c(20, 25, 30, 35, 40, 45, 50),

init.type = 'Spectral',

prevalence =~ room_type + s(date_number) + room_type * s(date_number),

data = out$meta,

max.em.its = 1)

plot(storage_less)

plot(storage_less$results$semcoh,

storage_less$results$exclus,

xlab= "Semantic coherence",

ylab= "Exclusivity",

col= "blue",

pch = 19,

cex = 1,

lty = "solid",

lwd = 2)

text(storage_less$results$semcoh,

storage_less$results$exclus,

labels=storage_less$results$K,

cex= 1,

pos=2)

```

## 1.5 Interpret

```{r}

labelTopics(model_k40, n = 10)

```

```{r}

# See Representative Text

thoughts_40 <- findThoughts(model_k40,

texts = thoughts_text,

topics = c(40),

n = 15)

plot(thoughts_40,width = 190)

```

```{r}

# See the Influence of Covariates

out$meta$room_type = as.factor(out$meta$room_type)

class(out$meta$date_number)

effect_k40 = estimateEffect(formula = 1:40 ~ room_type + s(date_number) + room_type * s(date_number),

stmobj = model_k40,

metadata = out$meta,

uncertainty = "Global")

summary(effect_k40)

plot(effect_k40, covariate = "room_type", topics = 10)

```

## 1.6 Visualize

```{r}

plot(model_k40, type = "summary",

n = 5, xlim = c(0, 0.2))

# custom proportion extraction

proportions_table <- make.dt(model_k40)

summarize_all(proportions_table, mean)

```

```{r}

plot(effect_k40, covariate = "room_type", topics = 21,

model = model_k40, method = "difference",

cov.value1 = "Entire home/apt", cov.value2 = "Private room",

xlab = "Private room ...... Entire home/apt",

labeltype = "custom", custom.labels = 21,

main = "Effect of Private room vs. Entire home/apt",

xlim = c(-0.05, 0.05))

```

```{r}

# One Line

plot(effect_k40, covariate = "date_number",

topics = 32, method = "continuous",

xaxt = "n", xlab = "Time (2020)", ylab = "Topic proportion", ylim = c(0,0.02), printlegend = FALSE)

monthnames <- c("Jan","Feb","Mar","Apr","May","Jun","Jul","Aug","Sep","Oct","Nov","Dec")

axis(1, at = as.numeric(monthseq) - min(as.numeric(monthseq)),

labels = monthnames)

```

```{r}

# Two Lines modified

model_k40_interact = estimateEffect(formula = c(32) ~ room_type * s(date_number),

stmobj = model_k40,

metadata = out$meta,

uncertainty = "None")

plot(model_k40_interact, covariate = "date_number", model = model_k40,

method = "continuous",xaxt = "n", xlab = "Time (2020)", ylab = "Topic proportion",

moderator = "room_type",

moderator.value = "Private room", linecol = "black",

ylim = c(0,0.048), ci.level = 0, printlegend = FALSE)

plot(model_k40_interact, covariate = "date_number", model = model_k40,

method = "continuous",xaxt = "n", xlab = "Days", moderator = "room_type",

moderator.value = "Entire home/apt", linecol = "palegreen",

add = TRUE, ci.level = 0, printlegend = FALSE)

monthnames <- c("Jan","Feb","Mar","Apr","May","Jun","Jul","Aug","Sep","Oct","Nov","Dec")

axis(1, at = as.numeric(monthseq) - min(as.numeric(monthseq)),

labels = monthnames)

legend(0,0.048, c("Private Room","Entire Place"), lty = c(1,1),

lwd = c(2,2), col = c("black","palegreen"))

```

```{r}

cloud(model_k40,32)

```

```{r}

# See the Relation among Topics

options(max.print = 2000)

cormat <- topicCorr(model_k40)

plot(cormat,

vlabels = c(1:40),

vertex.color = "#99ffcc",

vertex.label.cex = 1.2)

```

```{r}

checkBeta(model_k40)

```

```{r}

make.dt(model_k40)

```
